# Identifying and sequencing a *Mycobacterium sp*. strain F4 as a potential bioremediation agent for quinclorac

**DOI:** 10.1371/journal.pone.0185721

**Published:** 2017-10-02

**Authors:** Yingying Li, Wu Chen, Yunsheng Wang, Kun Luo, Yue Li, Lianyang Bai, Feng Luo

**Affiliations:** 1 College of Plant Protection, Hunan Agricultural University, Changsha, China; 2 School of Computing, Clemson University, Clemson, United States of America; 3 Hunan Academy of Agricultural Sciences, Changsha, China; Hubei University, CHINA

## Abstract

Quinclorac is a widely used herbicide in rice filed. Unfortunately, quinclorac residues are phytotoxic to many crops/vegetables. The degradation of quinclorac in nature is very slow. On the other hand, degradation of quinclorac using bacteria can be an effective and efficient method to reduce its contamination. In this study, we isolated a quinclorac bioremediation bacterium strain F4 from quinclorac contaminated soils. Based on morphological characteristics and 16S rRNA gene sequence analysis, we identified strain F4 as *Mycobacterium sp*. We investigated the effects of temperature, pH, inoculation size and initial quinclorac concentration on growth and degrading efficiency of F4 and determined the optimal quinclorac degrading condition of F4. Under optimal degrading conditions, F4 degraded 97.38% of quinclorac from an initial concentration of 50 mg/L in seven days. Our indoor pot experiment demonstrated that the degradation products were non-phytotoxic to tobacco. After analyzing the quinclorac degradation products of F4, we proposed that F4 could employ two pathways to degrade quinclorac: one is through methylation, the other is through dechlorination. Furthermore, we reconstructed the whole genome of F4 through single molecular sequencing and *de novo* assembly. We identified 77 methyltransferases and eight dehalogenases in the F4 genome to support our hypothesized degradation path.

## Introduction

Bioremediation using microorganisms is an important method to reduce the contamination of toxic chemical residue [1]. Due to the low dosage, longevity and especially effective control of barnyard grass and certain dicot grasses, quinclorac (3,7-dichloro-8-quinoline-carboxylic acid) is widely used in rice fields [2]. The quinclorac causes the cyanide accumulation in the tissues of susceptible plants, and then eventually leads to their death [2,3]. The quinclorac residues are phytotoxic to many crops and vegetables. In fields of rice-economic crops and vegetables rotation, the phytotoxicity of quinclorac can significantly reduce the yield and quality of economic crops and vegetables, such as rice and tobacco [4]. Meanwhile, due to its high stability, the degradation of quinclorac is very slow [5]. Currently, the major methods used to reduce quinclorac contamination are photodegradation and microbial degradation [6,7]. Photodegradation is only useful when quinclorac exists on the soil surface while microbial degradation is not limited by this condition. Several microbes have already been identified for quinclorac degradation. Lü et al. isolated the WZ1 strain from pesticide altered soil that is capable of degrading quinclorac [8]. Lü et al. identified the WZ1 strain as *Burkholderia cepacia* [8]. At optimal conditions, WZ1 could degrade 90% of quinclorac from an initial concentration of 1000 mg/L in 11 days [8,9,10]. In 2012, Xu et al. isolated a *Bordetella sp*. strain HN36 [11] from pesticide altered soil. The HN36 strain could degrade 96% of quinclorac from an initial concentration of 400 mg/L in 48 h [11]. In 2013, Dong et al. isolated a quinclorac degrading J3 strain from soil with long-term quinclorac usage and identified this strain as *Alcaligenes sp* [12], The J3 strain could degrade 70% of quinclorac (50 mg/L quinclorac) in seven days under optimal conditions [12]. Fan et al. isolated a QC06 *Pantoea* sp. strain from soil with long-term quinclorac usage in 2013, which could degrade 95.31% of quinclorac (50 mg/L) in seven days under optimal degrading conditions [13]. Liu et al. [14] isolated an endophytic *Bacillus megaterium* Q3 strain from roots of tobacco that had been grown in a quinclorac contaminated field. Q3 degraded 93.6% of the quinclorac in seven days under optimal conditions.

In this paper, we identified a *Mycobacterium sp*. strain F4 as a new bioremediation agent for quinclorac. We investigated the morphological, physiological, degradation characteristics and optimal degradation condition of F4. Moreover, we conducted an indoor pot experiment to demonstrate that the quinclorac degradation products of F4 are non-phytotoxic to tobacco. We analyzed the quinclorac degradation products of F4 using HPLC-MS/MS. We propose that F4 employs two pathways to degrade quinclorac. One transforms quinclorac to quinclorac methyl ester; the other transforms quinclorac to 3-chloro-7-hydroxyquinoline-8-carboxylic acid or 7-chloro-3-hydroxyquinoline-8-carboxylic acid. Furthermore, we determined the genome sequence of F4 using PacBio RSII single-molecule real-time (SMRT) sequencing. We reconstructed a single chromosome 6,103,712 in length and predicted 5,238 coding genes. We identified 77 methyltransferases and eight dehalogenases in the F4 genome to support our hypothesized degradation path.

## Materials and methods

### Materials

Quinclorac standard (98.1%) and other analytical reagents were bought from the China National Pharmaceutical Group Corporation, SINOPHARM. The quinclorac contaminated soil and tobacco rhizosphere soil were collected from private land in Liyutang Village of Yongxing Town, Chenzhou City, Hunan Province, China. The collection of soil did not involve endangered or protected species and was safe to the environment. No specific permission was needed. The GPS coordinates of the field are E112° 43 ^'^~ 113° 35^'^and N25°54^'^~ 26°29'.

The mineral salt medium (MS) and the Luria–Bertani’s (LB) medium used in experiments were prepared following [9].

### Methods

#### Isolation of quinclorac-degrading bacteria in soil

We collected a 5-g soil sample and mixed it with 95 mL of MS medium adding 50 mg·L^−1^ of quinclorac. We incubated it for seven days at 30°C on a shaker at 160 rpm. Then, we transferred the bacterial suspension to a new MS medium with 50 mg·L^−1^ quinclorac and incubated it for seven days. We repeated this process four times to continuously enrich the bacteria. After enrichment, we diluted the fluid on a plate of MS medium with 50 mg·L^−1^ quinclorac acid added. We cultured it in an incubator at 30°C for three days. Bacterial colonies with clear zones were selected as potential quinclorac-degrading bacteria.

### Measurement of quinclorac degradation

The degradation of quinclorac was monitored by high performance liquid chromatography (HPLC) (Agilent, Santa Clara, CA, USA) equipped with a SPD-20A UV detector and an Acchrom C18 column measuring 5 μm × 4.6 mm × 250 mm (Acchrom Co., LTD, Beijing, China). For each measurement, 1 mL degradation liquid was centrifuged at 12000 rpm for 5 min. Then, the supernatant was filtered through a 0.22 μm filter membrane and used for the HPLC experiments. Water (mixed with 0.2% acetic acid) with methanol (30/70, v/v) was used as an effluent with a flow rate of 0.8 mL/min for HPLC. The detections were performed at a wavelength of 240 nm with column temperature of 30°C. The injection volume was 10 μL.

#### Characterization of quinclorac-degrading strain F4

We determined the F4 strain according to Bergey’s Manual of Determinative Bacteriology. We first studied the morphological characteristics of strain F4. We streaked F4 on solid LB media to observe its colonial morphology. We cultured strain F4 in liquid LB media for 28 h and scanned the strain morphology using a scanning electron microscope. Then, we assessed basic physiological and biochemical characteristics of the strain.

#### Measurement of F4 Growth

F4 was cultivated in liquid LB culture to the middle of log phase (OD_600_nm = 0.82), then was inoculated into mineral salt medium with 50 mg·L^−1^ quinclorac. The growth of F4 was determined by the spectrophotometric method operated under 600 nm. First, 0.1 mL of culture liquid was transferred into a centrifuge tube and centrifuged at 2152 xg for three minutes. The cells were collected and washed with 0.3% sterile saline three times and fully resuspended in 1 mL 0.3% sterile normal saline. Then, OD_600_ value was determined by a spectrophotometer (Shimadzu, Japan). Each experiment was repeated three times.

#### Mass spectrum of degradation products

The degradation products of quinclorac were determined by an Agilent 1260 LC system coupled to a 6530 QTOF MS with electrospray ionization via Agilent Jet Stream Technology. The column used in this study was Uniraty C18 (5mm, 2.1mm×150mm) provided by Acchrom Co. Ltd, Beijing, China). For each measurement, 1 mL degradation liquid was centrifuged at 12000 rpm for five minutes. Then, the supernatant was filtered through a 0.22 μm filter membrane and used for LC experiments. The degradation products were determined according to the chromatographic peaks, and the degradation products were analyzed by mass spectrometry. The product was then scanned by a secondary mass spectrometry (MS/MS) and broken up to obtain the fragment ion information of the degradation products.

The aqueous constituent of the LC mobile phase was 0.2% formic acid in water (eluent A). The organic modifier of the LC mobile phase was 0.2 formic acid in acetonitrile (eluent B). A linear gradient of two eluents was optimized. Flow rate was set at 0.2 ml/min. The injection volume was 5 ml and the column temperature was maintained at 30°C in each run.

The mass spectrometer was operated on positive mode. The parameter settings used for the measurement were as follows: capillary voltage 4 kV, nozzle voltage 0 kV, nebulizer pressure 50 psi, dry gas 6 L/min, gas temperature 300°C, skimmer voltage 65 V, OCT1 RF Vpp at 750 V, with a fragmentor voltage 135 V. Data were acquired using the extended dynamic range mode (2 GHz) and collected in the full-scan mode from m/z 100 to 1500 in centroid mode. The TOF was calibrated every day before sample analysis using reference masses at m/z 112.988 and 1033.9881 (positive ion mode) to obtain high-accuracy mass measurements.

#### Pot experiment

The pot cultivation of tobacco was conducted under three conditions: 1) soil with 10 mg·L^−1^ quinclorac, 2) soil with seven-day degradation liquid of F4, and 3) soil with water. After cultured in liquid mineral salt medium, 200 mL F4 fermentation broth was centrifuged at 12000 rpm for 5 min to remove bacteria cells. Then, the degradation liquid was applied to the leaves and roots of tobacco in the pot for five days. Five replicate experiments were conducted in each condition.

We collected soil from the tobacco field of Hunan Agricultural University. No quinclorac was detected in the soil. We transplanted one tobacco seedling into each pot. The tobacco was cultured in a greenhouse after transplanting. Obvious phytotoxic symptoms were observed within approximately 25 days after transplanting. Then, we measured leaf length, leaf width, and height of each tobacco plant.

#### F4 genome sequencing, assembly and annotation

The genome sequence of F4 was determined by PacBio RSII single-molecule real-time (SMRT) sequencing (Pacific Biosciences, Menlo Park, CA, USA). The genomic DNA of the F4 strain was extracted by QIAamp genomic DNA kits (Qiagen, Hilden, Germany). The concentration and quality of the genomic DNA were determined by Agilent 2100 Bioanalyzer (Palo Alto, CA, USA). The whole genome was assembled using the RS Hierarchical Genome Assembly Process 2.3 in the SMRT analysis program 2.3 (Pacific Biosciences) [15] and genome annotation was carried out using the Prokaryotic Genome Annotation Pipeline 4.0 of NCBI [16].

## Results

### Identification and characterization of a quinclorac degrading bacteria, *Mycobacterium sp*. F4

After culturing quinclorac contaminated soil, we isolated three bacteria strains with clear zone colonies, which had the capability to degrade quinclorac. Among them, strain F4 had the best degradation rate and was chosen for subsequent study.

**Fig 1:**
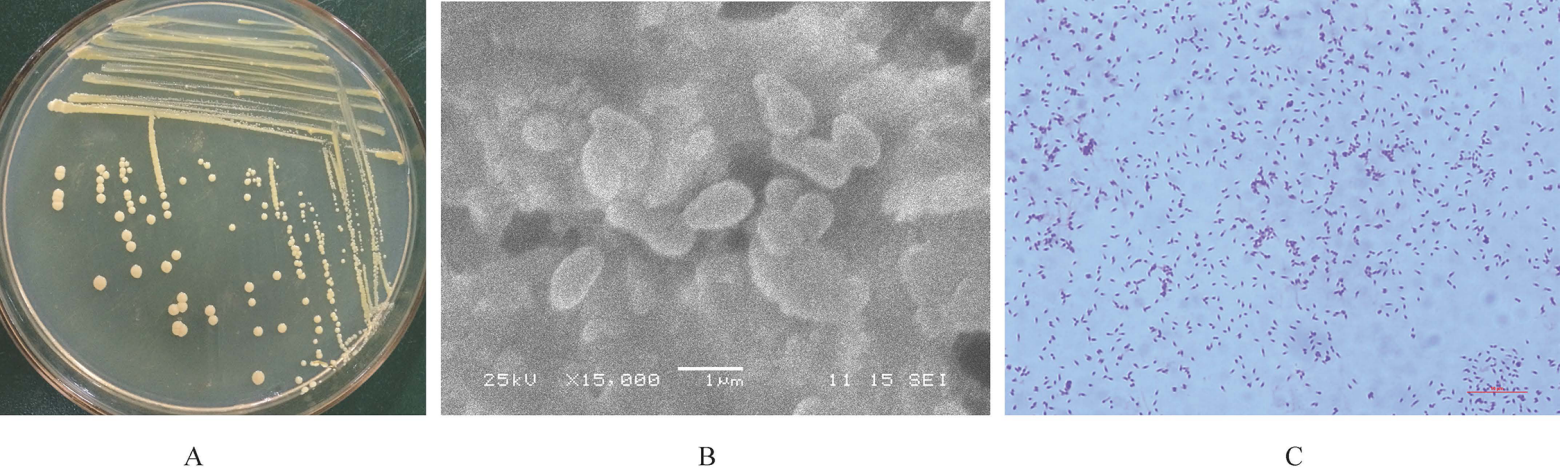
Colony morphology (A), cell morphology (B), and gram staining (C) of F4.

We first studied the morphological characteristics of F4. After being cultured with LB medium for 4 days, the colonial morphology of F4 on LB plates was circular, convex, smooth, and faint yellow (Fig 1A). The cell morphology of F4 under a scanning electron microscope was a small ellipsoid and 0.5–0.7 × 1 μm in size, occurred singly or in groups (Fig 1B). Then, we assessed the basic physiological and biochemical characteristics of F4. Gram staining tests showed that F4 is a Gram-positive bacterium (Fig 1C). Both Kolmer and Indole tests were negative.

### Determination of optimal degradation conditions of F4

#### Effect of inoculation size on degradation of quinclorac

**Fig 2:**
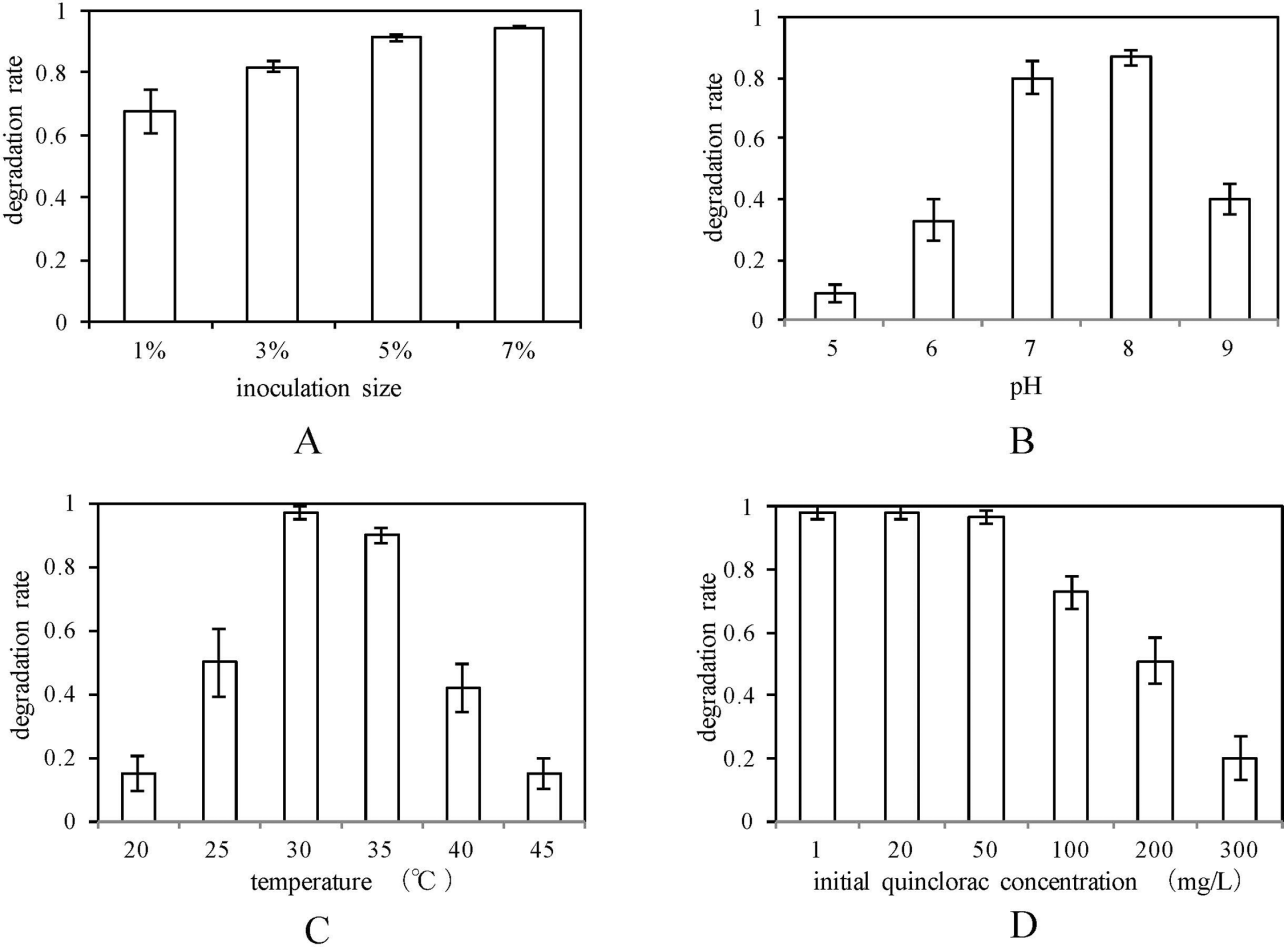
Effect of temperature (A), pH (B), inoculation size (C), and initial quinclorac concentration (D) on degradation of quinclorac and growth of F4.

To study the effect of inoculation amounts of F4, we inoculated 1%, 3%, 5%, 7% (volume ratio) of F4 into degradation medium, respectively. Then, we measured the degradation rate of quinclorac after seven days of incubation. Fig 2A shows the effect of inoculation size on quinclorac degradation rates of F4; thus, after increasing the inoculation size from 1% to 5%, the quinclorac degradation rates of F4 increased. The degradation rate reached a peak value of 97% at an inoculation of 5%.

#### Effect of pH on degradation of quinclorac

We set the pH of degradation culture media to 5, 6, 7, 8, and 9, respectively, and incubated the five F4 cultures for seven days. Then, we measured the quinclorac degradation rate of F4. Fig 2B plotted the quinclorac degradation rate of F4 against pH. It shows that the F4 culture media reached the highest degradation rate at pH 8 level, which was 86%. The degradation of quinclorac sharply decreased when the culture medium was set to both low and high pH levels. Weak alkaline conditions allow better utilization of quinclorac, which is weakly acidic, while it does not affect the growth of F4.

#### Effect of temperature on degradation of quinclorac

We incubated the F4 with degradation culture media at 20°C, 25°C 30°C, 35°C, 40°C, and 45°C separately. We measured the degradation rate of quinclorac and growth of F4 after seven days of incubation. Fig 2C plotted the degradation rate of quinclorac against temperature. It shows that temperature had a significant effect on degradation of quinclorac using F4. When the culture temperature was increased from 15°C to 30°C, growth of F4 and degradation of quinclorac also increased. At the temperature of 30°C, the degradation rate of quinclorac reached peak values, which were 97%. When the temperature was increased above 30°C, the degradation rate of quinclorac decreased.

#### Effect of initial quinclorac concentration on degradation of quinclorac

To study the effect of initial quinclorac concentration on quinclorac degradation of F4, we incubated F4 on different degradation media with initial quinclorac concentrations of 1, 20, 50, 100, 200, and 300 mg/L. Then, we measured the quinclorac degradation rate of F4 after 7 days. Fig 2D shows that the quinclorac degradation rates of F4 remained high when initial quinclorac concentration was less than 50 mg/L. However, F4 could not grow well when the initial quinclorac concentration reached 100mg/L; the degradation rate reduced dramatically when the initial quinclorac concentration was above 100 mg/L.

### Relationship between F4 growth and the quinclorac degradation

**Fig 3:**
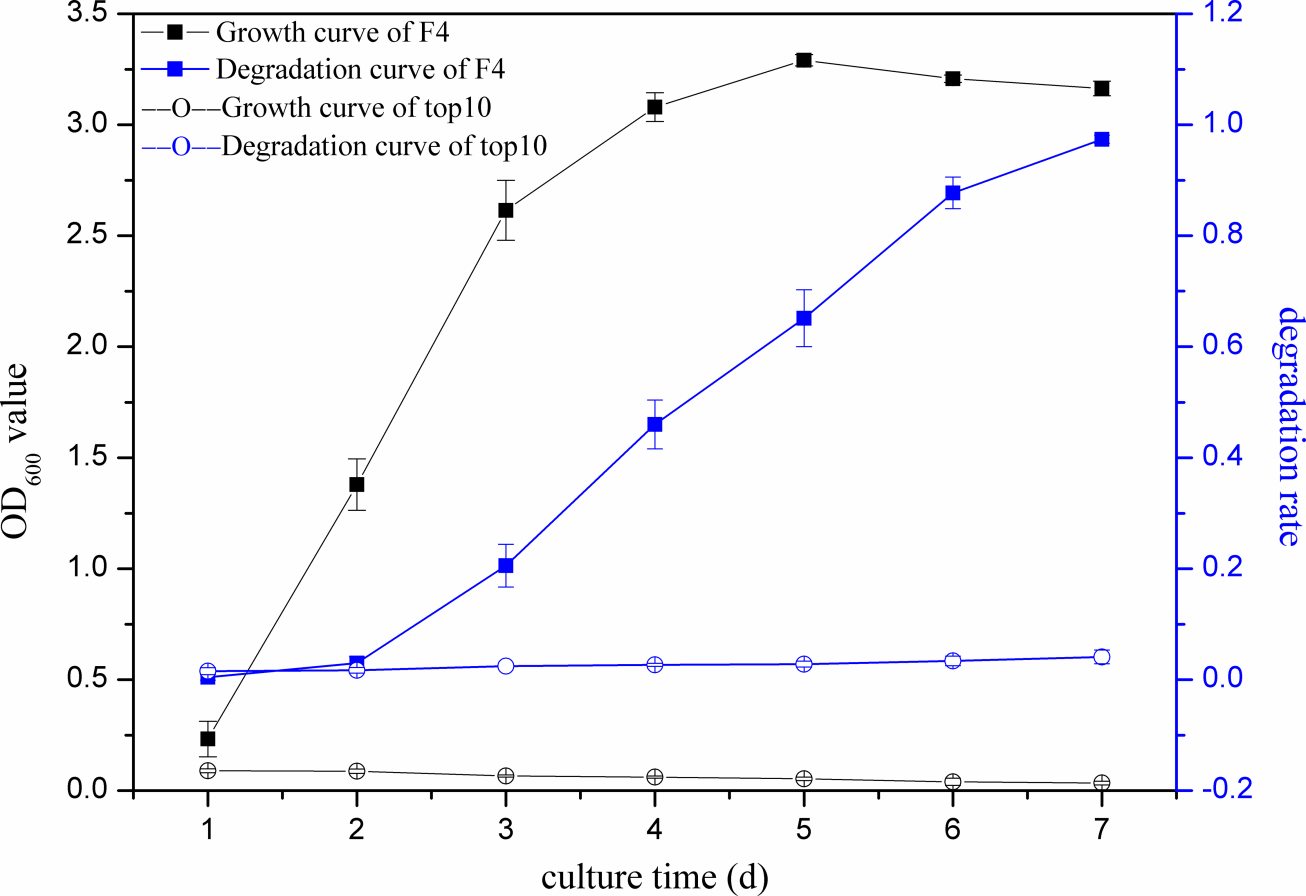
Growth curve and degradation curve of F4 and *E coli* Top10 (control) after different culture timed durations.

We incubated F4 under optimal conditions: temperature at 30°C, pH at 8, with a 6% inoculation size, and initial quinclorac concentration at 50 mg/L. We measured the degradation rate of quinclorac and growth of F4 for seven days. Fig 3 shows that the quinclorac was not degraded in the first two days and was slightly degraded on the third day. Then, the degradation rate increased sharply on the fourth, fifth and sixth days. After that, the degradation rate only slightly increased. The degradation rate on the seventh day was 97.38%. Meanwhile, OD_600_ values showed that F4 grew dramatically in the first two days; then, F4 grew gradually and reached its peak value by the fifth day when growth of F4 began to decline. As a control, we also measured the growth and degradation curve of *E*. *coli* Top10 strain. Fig 3 shows that *E*. *coli* Top10 barely grew in MS media with 50 mg/L quinclorac and its degradation rate was closed to zero. This comparison demonstrated the effectiveness of F4 for quinclorac degradation.

### Analysis of quinclorac degradation products of F4

**Fig 4:**
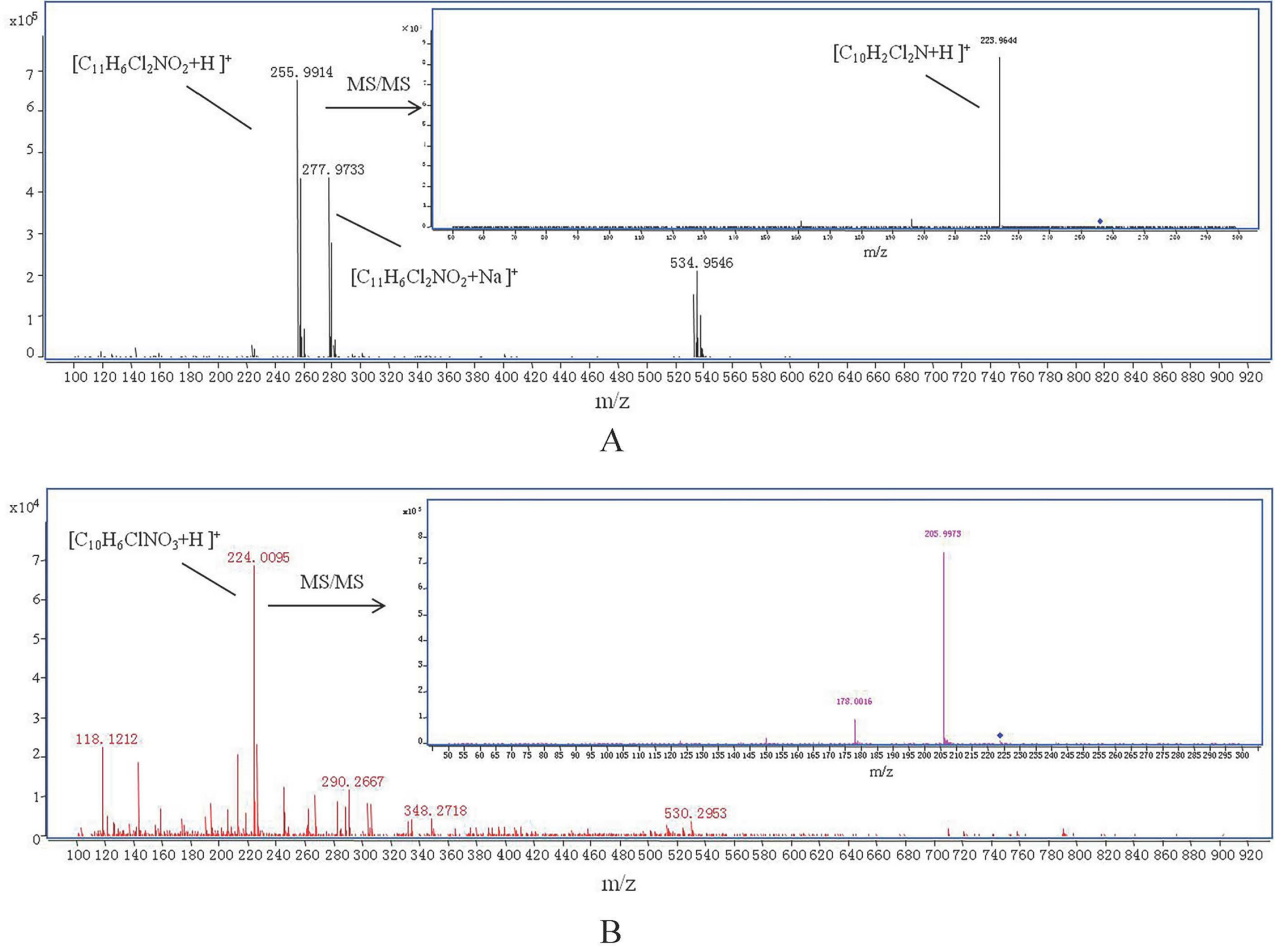
Mass spectrum of two quinclorac degradation products by F4: quinclorac methyl ester (A) and 3-chloro-7-hydroxyquinoline-8-carboxylic acid or 7-chloro-3-hydroxyquinoline-8-carboxylic acid (B). Inner figures are secondary mass spectrum.

**Fig 5:**
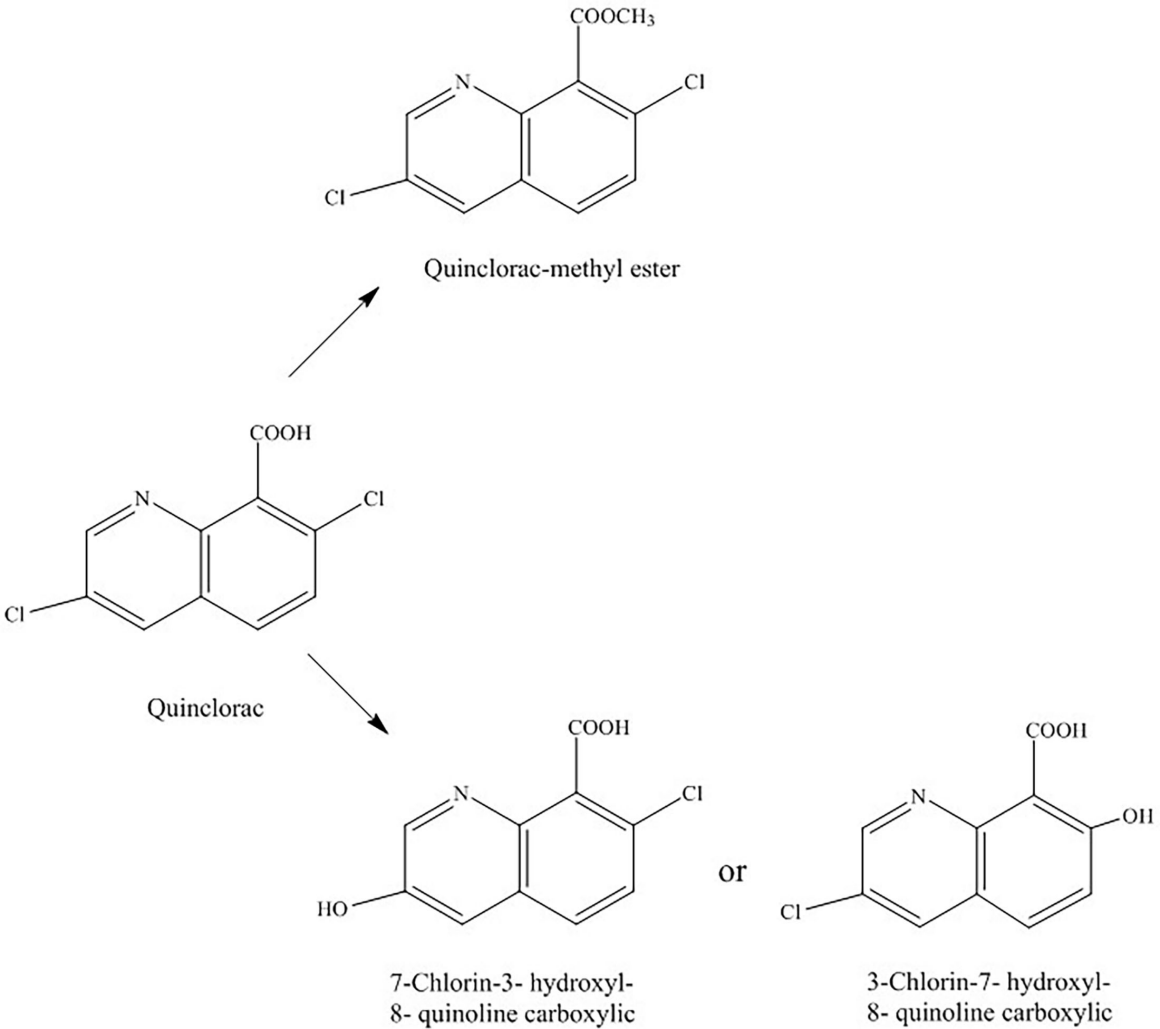
Possible F4 quinclorac degradation products and pathways.

We isolated the degradation products of quinclorac and analyzed those using LC-MS/MS. Two degradation products were detected after F4 was incubated in degradation media for 4 days. Fig 4A shows the mass spectrum of the first degradation product, which is the major degradation product. The major peak was at 255.9914 *m/z*, which was resolved as quinclorac methyl ester. The peak at 277.9733 *m/z* can be resolved as quinclorac methyl ester+Na, which further confirmed the first product as quinclorac methyl ester. The second mass spectrum has one peak at 223.9644 *m/z*. Fig 4B shows the mass spectrum of the second degradation product. The peak at 224.0095 *m/z* was resolved as 3-chloro-7-hydroxyquinoline-8-carboxylic acid or 7-chloro-3-hydroxyquinoline-8-carboxylic acid. We cannot distinguish which one is the actual degradation product based on mass spectrum only. The second mass spectrum had one peak at 205.9973 *m/z*. Based on the results of mass spectrum, we hypothesized that F4 may employ two pathways to degrade quinclorac: one is through methylation, the other is through dechlorination (Fig 5).

### Non-phytotoxicity of F4 quinclorac fermentation broth to tobacco

**Fig 6:**
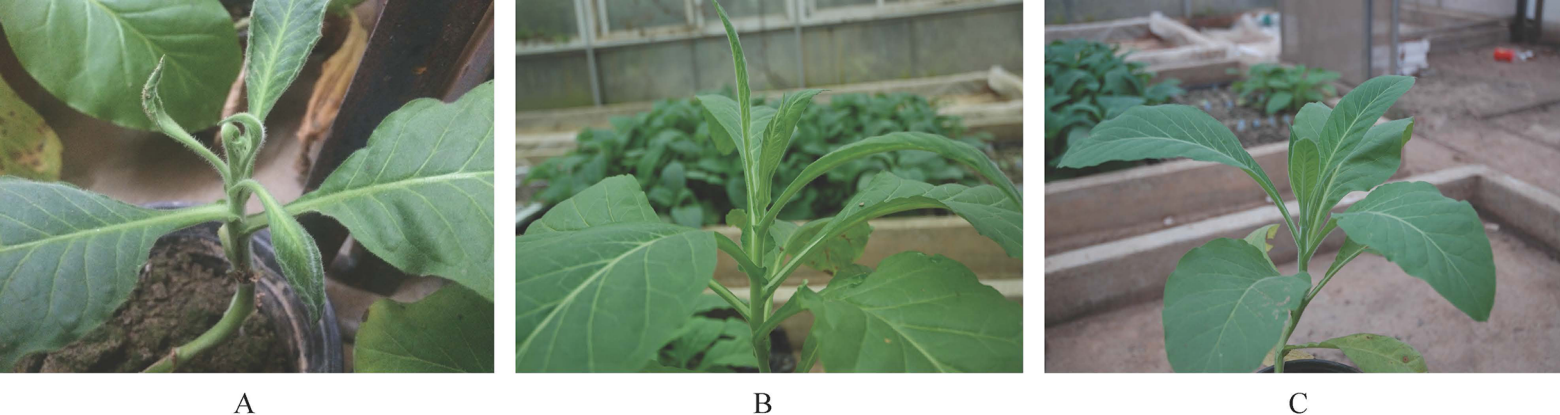
Phytotoxicity of quinclorac on tobacco (A), non-phytotoxicity of F4 quinclorac fermentation broth of F4 (B) and control (C).

To verify the non-phytotoxicity of an F4 quinclorac fermentation broth, we compared the growth of tobacco in pots under different conditions: soil with quinclorac added; soil with seven days of F4 fermentation broth added; and negative controls with only water added to the soil. Table 1 compares the leaf length, leaf width and plant height of tobacco in soils under three conditions. The results showed that the F4 quinclorac fermentation broth had non-phytotoxicity to tobacco. After transplanting for 25 days, in the soil with quinclorac (10 mg/L), the leaf length, leaf width and plant height of tobacco were all significantly inhibited, which were only 44%, 71% and 40% of those representing the control. In contrast, in soil with seven days F4 quinclorac fermentation broth, leaf length, leaf width and plant height of tobacco were 105%, 96% and 97% of those representing the control. Fig 6 shows the growth of tobacco under different conditions after 25 days.

**Table 1:** Effects of non-phytotoxic F4 quinclorac fermentation broth on leaf length, leaf width and plant height of tobacco.

	10 ml/L quinclorac (cm)	7 days F4 quinclorac fermentation broth (cm)	water (cm)
Leaf length	8.33±1.49	20±1.63	18.917±1.54
Leaf width	5.467±0.76	7.33±1.07	7.667±0.94
Plant height	8.833±3.18	21.4±3.17	22±3.58

Note: Values are the means ±standard deviation of five replicates.

### Whole genome sequence of F4

**Fig 7:**
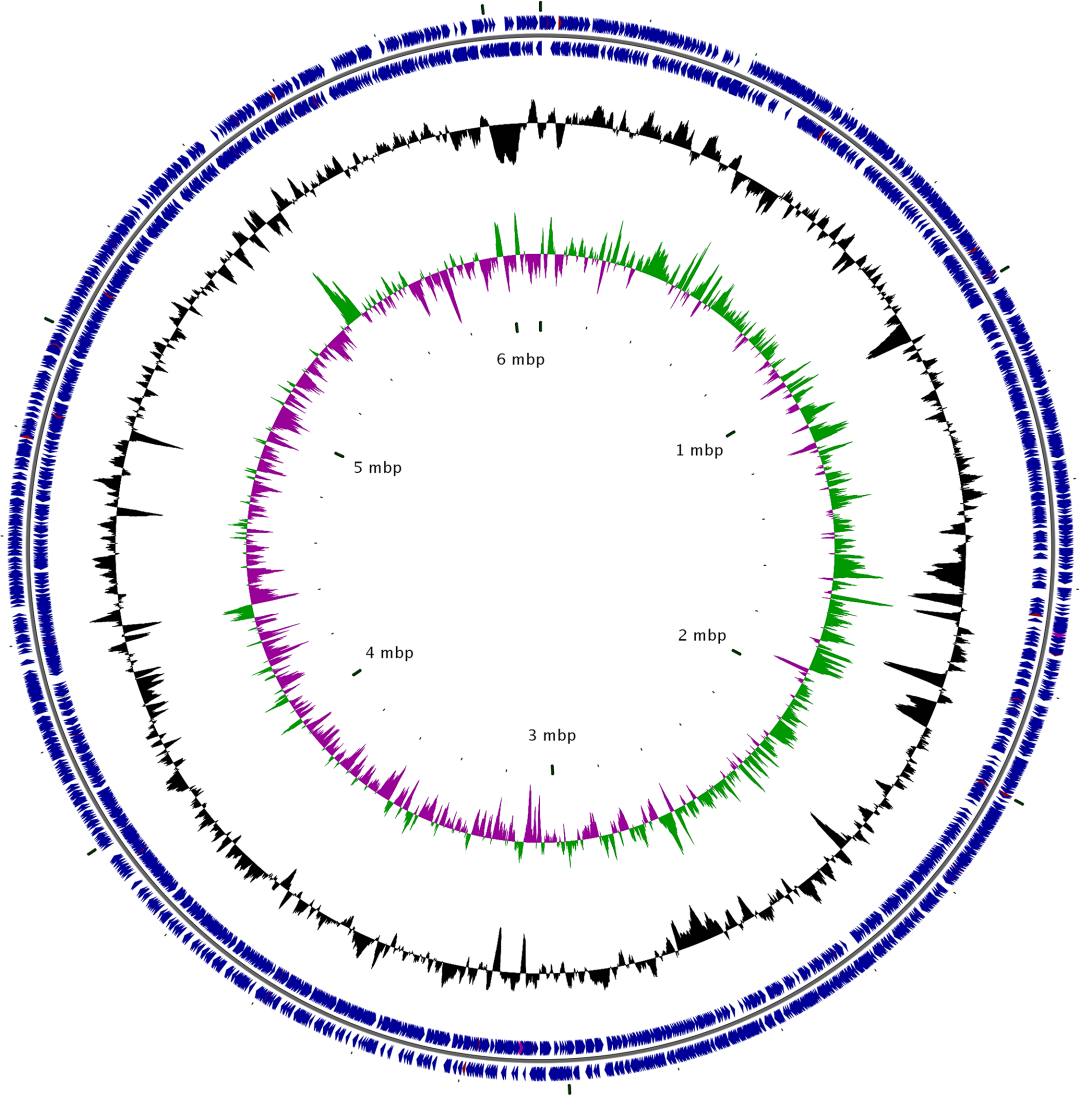
Circular genome map of F4. Outer ring represents genes; the middle ring represents average GC content, and the inner ring represent GC skew.

We sequenced the genome of F4 using PacBio SMRT sequencing technology. We obtained 173,187 reads and a total of 1,433,680,516 base pairs with a mean read length of 8,278 bp. We assembled a single F4 chromosome 6,103,712 bp in length (Fig 7). The G+C content of the F4 genome is 67.35%. From this F4 genome, we predicted 5,238 coding genes, 47 tRNA genes, 3 ncRNAs, 6 rRNAs and 545 pseudogenes. Out of 5,238 coding genes, 3,216 could find COG orthologs with a minimum amino acid similarity of 75%.

We identified two complete rRNA clusters (16S-23S-5S rRNA) in F4 genomes and the two 16S rRNAs were 100% identical. We then aligned the F4 16S rRNA gene sequences on the EzTaxon Server 2.1, which revealed that the closest species related to the F4 strain were *Mycobacterium pallens* (accession number DQ370008) and *Mycobacterium senegalense* (accession number AY457081), with a similarity of 99.44% and 99.38%, respectively.

**Fig 8:**
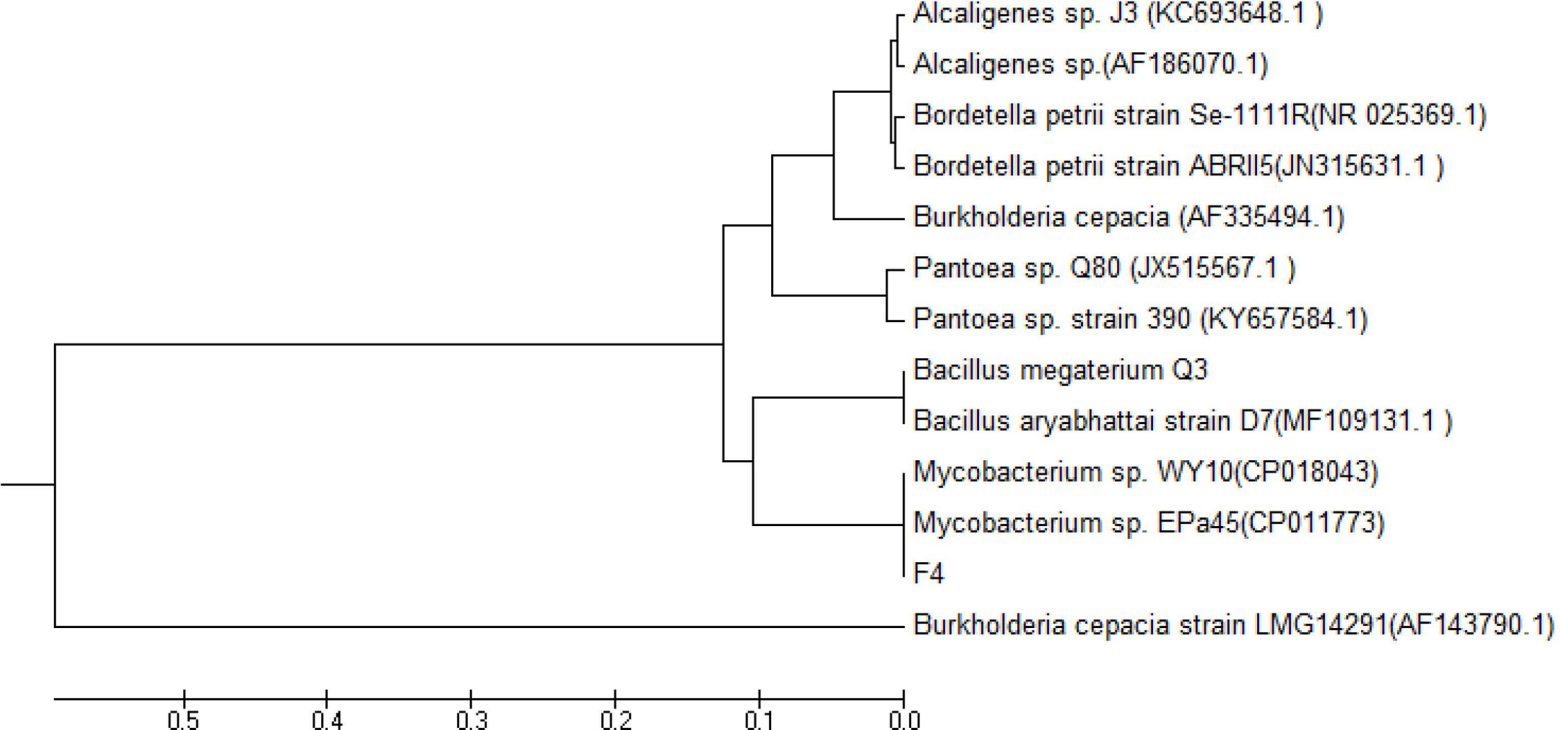
The phylogenetic tree analysis of strain F4.

We then aligned the 16s rRNA sequence of F4 with other two Mycobacterium and several herbicide degrading bacteria and constructed a phylogenetic tree (Fig 8). The phylogenetic analysis clearly showed that the F4 strain belongs to the same branch of Mycobacterium bacteria and was obviously different from other quinclorac degrading bacteria, such as *Burkholderia cepacia* [8] *Bordetella petrii* [11], *Alcaligenes sp* [12], and *Pantoea* sp [13]. Therefore, we identified and designated the isolated strain of F4 as Mycobacterium *sp*.

### Comparative genome analysis of F4, *Mycobacterium* sp. EPa45 and *Mycobacterium* sp. WY10

**Fig 9:**
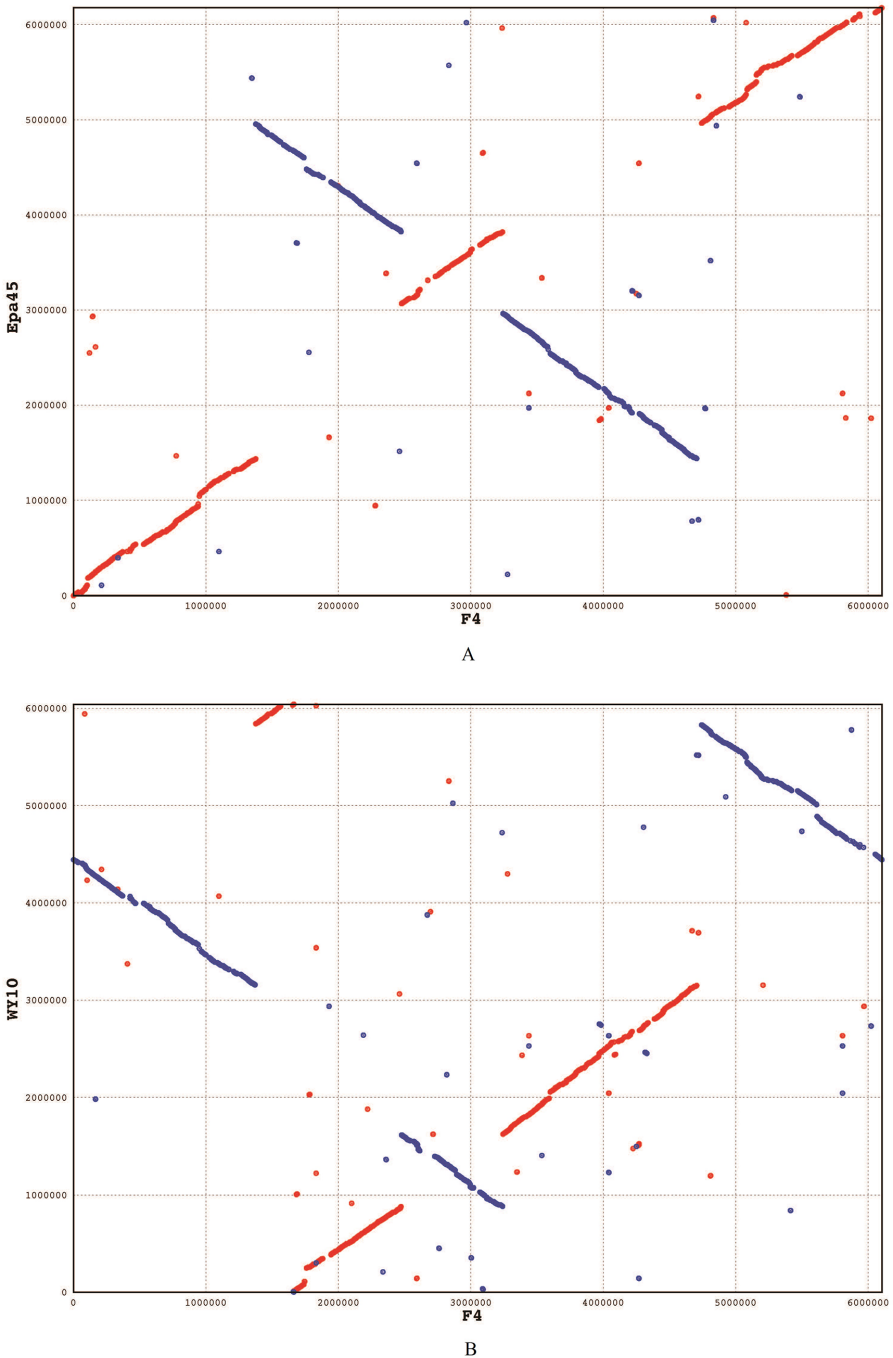
Whole genome alignment of genome F4 against EPa45 (A) and WY10 (B).

To further understand the F4 genome, we compared it to two closely related genomes of F4, *Mycobacterium* sp. EPa45 and *Mycobacterium* sp. WY10. We first performed whole genome alignment using LASTZ Release 1.04.00 [17] with chain and MUMmer (version 3.23) [18]. We used F4 as the target genome and aligned EPa45 and WY 10 with it (Fig 9). There were 638 matches with a length of more than 1000 base pairs between the F4 and Epa45 strain, corresponding to a total of 4,439,508 bp in the target genome (F4) with a minimum identity of 59.2%. Similarly, there were 495 matches with a length of more than 1000 base pairs between F4 and WY10, corresponding to a total of 3,437,020 bp in the target genome (F4) with a minimum identity of 60.9%. Among these matched segments, 3,360,698 base pairs were overlapped with each other. As shown in Fig 9, there are genome arrangements between F4 and WY10, as well as between F4 and EPa45.

We then compared the gene families in three genomes. The gene families were constructed using OrthoMCL with inflation index of 1.5 [19]. The F4, EPa45 and WY10 genomes contain 5,783, 5,822 and 5,599 predicted proteins, respectively. And we identified as 4,213, 4,975 and 4,852 gene clusters in F4, EPa45 and WY10 genomes respectively. The F4 genome has more singleton genes (1,393) compared to those in EPa45 (666 genes) and WY10 (649 genes) genomes. Totally, there are 5,143 orthologous clusters (which contain at least two species). Among them, 3,599 orthologous clusters have members from all three genomes; moreover, 3,522 out of 3,599 clusters were single-copy gene clusters. The biggest cluster contains 27 members, nine genes from each organism. The genes in this cluster belong to the MmpL family RND transporter, which plays key roles in exporting cell-wall associated lipids and siderophores in mycobacteria [20,21].

### Methyltransferases and dehalogenases in F4 genome

After analyzing the quinclorac degradation products of F4, we hypothesized that F4 degraded quinclorac through methylation and dechlorination. To verify the possibility of those degradation paths, we determined if the F4 genome contains methyltransferase, which facilitates the first transformation, and dehalogenases, which helps the second transformation. In total, we identified 77 methyltransferases in the F4 genome (S1 Table), and 44 out of 77 are annotated as S-adenosyl methionine (SAM)-dependent methyltransferase. S-adenosyl methionine (SAM) is a universal biological cofactor that is found in all branches of life where it plays a critical role in transferring methyl groups to various biomolecules, including DNA, proteins and small-molecule secondary metabolites. Meanwhile, we also identified eight genes in F4 genomes that belong to the dehalogenases family (S2 Table). Those results implied that our hypothesized degradation pathways may be possible in F4.

## Discussion

In this study, we have isolated and identified a quinclorac-degrading bacterium strain F4 that can effectively degrade quinclorac at low concentration. Two degradation products by F4 were identified as quinclorac methyl ester (major), 3-chloro-7-hydroxyquinoline-8-carboxylic acid or 7-chloro-3-hydroxyquinoline-8-carboxylic acid (minor). The quinclorac methyl ester was previously reported as one of degradation products of quinclorac [22]. Our indoor pot experiments showed that the F4 quinclorac fermentation broth was non-phytotoxic to tobacco. A previous study also showed quinclorac methyl ester has very little inhibition of shoot growth for leafy spurge [23]. Furthermore, we reconstructed the whole genome sequence of F4 and predicted 77 methyltransferases and eight dehalogenases in the F4 genome. These results showed that F4 may employ two degradation pathways; one transforms quinclorac to quinclorac methyl ester through methylation and the other transforms quinclorac to 3-chloro-7-hydroxyquinoline-8-carboxylic acid or 7-chloro-3-hydroxyquinoline-8-carboxylic acid through dechlorination. However, the key enzymes of the F4 genome in the process of quinclorac degradation remain unknown. We will elucidate the enzymes in F4 for quinclorac degradation and the transcription factors that control the quinclorac degradation in future research.

## Supporting information

S1 Table List of methyltransferase genes in F4. (DOCX)

S2 Table List of dehalogenases genes in F4. (DOCX)
