# Supplementary material for: Identifying and sequencing a Mycobacterium sp. strain F4 as a potential bioremediation agent for quinclorac
Source: PLoS One. 2017 Oct 2;12(10):e0185721. doi: 10.1371/journal.pone.0185721 (PMC5624592; doi:10.1371/journal.pone.0185721)
Supplement: S1 Table — (DOCX) [file pone.0185721.s001.docx]

**S1 Table 1. List of methyltransferase genes in F4**

| GeneID | Location | Annotation |
| --- | --- | --- |
| B1R94_00455 | CP019882:81816:82451:- | SAM-dependent methyltransferase; |
| B1R94_00810 | CP019882:157361:158242:- | SAM-dependent methyltransferase; |
| B1R94_00990 | CP019882:193314:194249:+ | SAM-dependent methyltransferase; |
| B1R94_01205 | CP019882:234471:235136:+ | methyltransferase; |
| B1R94_01360 | CP019882:271011:271769:- | tRNA (guanosine(46)-N7)-methyltransferase TrmB; |
| B1R94_01505 | CP019882:305812:306590:- | SAM-dependent methyltransferase; |
| B1R94_02500 | CP019882:537678:538577:+ | SAM-dependent methyltransferase; |
| B1R94_02515 | CP019882:541760:542530:+ | trans-aconitate methyltransferase; |
| B1R94_03070 | CP019882:652782:653450:+ | RNA methyltransferase; |
| B1R94_03570 | CP019882:754823:755728:- | SAM-dependent methyltransferase; |
| B1R94_03805 | CP019882:803025:803900:+ | SAM-dependent methyltransferase; |
| B1R94_03860 | CP019882:811463:812269:- | SAM-dependent methyltransferase; |
| B1R94_04270 | CP019882:888148:888777:+ | SAM-dependent methyltransferase; |
| B1R94_04720 | CP019882:969844:970698:- | SAM-dependent methyltransferase; |
| B1R94_04725 | CP019882:970780:971715:- | SAM-dependent methyltransferase; |
| B1R94_04730 | CP019882:971823:972371:+ | SAM-dependent methyltransferase; |
| B1R94_04985 | CP019882:1024398:1025333:- | SAM-dependent methyltransferase; |
| B1R94_04990 | CP019882:1025429:1026313:- | SAM-dependent methyltransferase; |
| B1R94_05540 | CP019882:1136298:1137215:- | SAM-dependent methyltransferase; |
| B1R94_05545 | CP019882:1137224:1138114:- | SAM-dependent methyltransferase; |
| B1R94_05550 | CP019882:1138114:1139043:- | SAM-dependent methyltransferase; |
| B1R94_05555 | CP019882:1139048:1139950:- | SAM-dependent methyltransferase; |
| B1R94_05560 | CP019882:1139947:1140867:- | SAM-dependent methyltransferase; |
| B1R94_06315 | CP019882:1287410:1288876:+ | serine hydroxymethyltransferase; |
| B1R94_06470 | CP019882:1319096:1319794:- | methyltransferase; |
| B1R94_06635 | CP019882:1349893:1350528:+ | SAM-dependent methyltransferase; |
| B1R94_06660 | CP019882:1353937:1354763:- | methyltransferase; |
| B1R94_06810 | CP019882:1386731:1387797:- | SAM-dependent methyltransferase; |
| B1R94_07020 | CP019882:1428702:1429367:- | methyltransferase; |
| B1R94_07615 | CP019882:1580504:1581616:+ | methyltransferase; |
| B1R94_07620 | CP019882:1581901:1582596:+ | rhamnosyl O-methyltransferase; |
| B1R94_07920 | CP019882:1669868:1670803:+ | protein-(glutamine-N5) methyltransferase, |
| B1R94_08010 | CP019882:1689036:1689542:- | cysteine methyltransferase; |
| B1R94_09360 | CP019882:2002051:2002935:- | homocysteine S-methyltransferase; |
| B1R94_09980 | CP019882:2138680:2139435:+ | 16S rRNA (uracil(1498)-N(3))-methyltransferase; |
| B1R94_10445 | CP019882:2234247:2234972:+ | ubiquinone biosynthesis methyltransferase UbiE; |
| B1R94_10480 | CP019882:2242051:2243139:- | SAM-dependent methyltransferase; |
| B1R94_10770 | CP019882:2299480:2300100:+ | SAM-dependent methyltransferase; |
| B1R94_11040 | CP019882:2358702:2359331:+ | SAM-dependent methyltransferase; |
| B1R94_11440 | CP019882:2440927:2441595:+ | methyltransferase; |
| B1R94_11640 | CP019882:2477791:2478663:+ | SAM-dependent methyltransferase; |
| B1R94_11785 | CP019882:2506995:2507750:+ | precorrin-4 C(11)-methyltransferase; |
| B1R94_12120 | CP019882:2574769:2575500:- | SAM-dependent methyltransferase; |
| B1R94_13035 | CP019882:2766809:2767633:+ | SAM-dependent methyltransferase; |
| B1R94_13175 | CP019882:2792014:2792616:+ | SAM-dependent methyltransferase; |
| B1R94_13430 | CP019882:2840968:2841852:- | SAM-dependent methyltransferase; |
| B1R94_13635 | CP019882:2881285:2881617:+ | RNA methyltransferase; |
| B1R94_14275 | CP019882:3018210:3019055:- | SAM-dependent methyltransferase; |
| B1R94_14495 | CP019882:3063955:3064551:+ | methyltransferase type 11; |
| B1R94_14615 | CP019882:3085513:3086321:- | 16S/23S rRNA (cytidine-2'-O)-methyltransferase; |
| B1R94_14825 | CP019882:3135779:3136579:- | RNA methyltransferase; |
| B1R94_16120 | CP019882:3419753:3421093:- | rRNA cytosine-C5-methyltransferase; |
| B1R94_16865 | CP019882:3567224:3567862:+ | SAM-dependent methyltransferase; |
| B1R94_17125 | CP019882:3622670:3623857:- | 23S rRNA methyltransferase; |
| B1R94_17920 | CP019882:3775739:3776950:- | uroporphyrinogen-III C-methyltransferase; |
| B1R94_18090 | CP019882:3811785:3812648:- | SAM-dependent methyltransferase; |
| B1R94_18160 | CP019882:3828932:3830022:- | 23S rRNA (adenine(2503)-C(2))-methyltransferase |
| B1R94_18185 | CP019882:3832999:3833679:- | SAM-dependent methyltransferase; |
| B1R94_18375 | CP019882:3880746:3881435:- | tRNA (guanosine(37)-N1)-methyltransferase TrmD; |
| B1R94_18560 | CP019882:3917875:3918426:- | 16S rRNA (guanine(966)-N(2))-methyltransferase |
| B1R94_19075 | CP019882:4025257:4026033:+ | SAM-dependent methyltransferase; |
| B1R94_19105 | CP019882:4031829:4033019:- | SAM-dependent methyltransferase; |
| B1R94_19110 | CP019882:4033016:4034014:- | SAM-dependent methyltransferase; |
| B1R94_20500 | CP019882:4309085:4309966:- | SAM-dependent methyltransferase; |
| B1R94_20680 | CP019882:4349877:4350623:- | SAM-dependent methyltransferase; |
| B1R94_20685 | CP019882:4350623:4351705:- | hydroxyneurosporene methyltransferase; |
| B1R94_21010 | CP019882:4424034:4424330:+ | DNA methyltransferase; |
| B1R94_21865 | CP019882:4596021:4596755:+ | SAM-dependent methyltransferase; |
| B1R94_22785 | CP019882:4793568:4794221:+ | methyltransferase; |
| B1R94_22940 | CP019882:4825618:4826373:+ | SAM-dependent methyltransferase; |
| B1R94_23205 | CP019882:4881908:4882669:+ | SAM-dependent methyltransferase; |
| B1R94_23960 | CP019882:5043285:5044141:- | rRNA methyltransferase; |
| B1R94_26485 | CP019882:5537665:5538414:+ | SAM-dependent methyltransferase; |
| B1R94_26495 | CP019882:5540019:5540699:+ | SAM-dependent methyltransferase; |
| B1R94_26505 | CP019882:5541818:5542783:- | L-histidine N(alpha)-methyltransferase; |
| B1R94_26695 | CP019882:5577081:5578397:+ | SAM-dependent methyltransferase; |
| B1R94_29170 | CP019882:6099946:6100649:- | 16S rRNA (guanine(527)-N(7))-methyltransferase |
