# Supplementary material for: Identifying and sequencing a Mycobacterium sp. strain F4 as a potential bioremediation agent for quinclorac
Source: PLoS One. 2017 Oct 2;12(10):e0185721. doi: 10.1371/journal.pone.0185721 (PMC5624592; doi:10.1371/journal.pone.0185721)
Supplement: S2 Table — (DOCX) [file pone.0185721.s002.docx]

**S2 Table 2. List of dehalogenase genes in F4.**

| GeneID | Location | Annotation |
| --- | --- | --- |
| B1R94_RS04335 | 900404:901093:- | haloacid dehalogenase |
| B1R94_RS04465 | 926754:927368:+ | haloacid dehalogenase, type II |
| B1R94_RS11965 | 2556661:2557473:+ | haloacid dehalogenase |
| B1R94_RS13255 | 2821217:2822158:+ | haloacid dehalogenase |
| B1R94_RS14180 | 3012828:3013514:+ | haloacid dehalogenase |
| B1R94_RS22175 | 4678202:4678939:- | haloacid dehalogenase |
| B1R94_RS23580 | 4976129:4976821:+ | haloacid dehalogenase, type II |
| B1R94_RS26760 | 5608900:5609802:- | haloalkane dehalogenase |
